# Stimulating Superior Cluneal Nerves via Peripheral Nerve Stimulation as a Treatment for Chronic Low Back Pain

**DOI:** 10.7759/cureus.51952

**Published:** 2024-01-09

**Authors:** Peter D Vu, Christopher L Robinson, Alan D Kaye, Jamal Hasoon

**Affiliations:** 1 Physical Medicine and Rehabilitation, University of Texas Health Science Center at Houston McGovern Medical School, Houston, USA; 2 Anesthesiology, Critical Care and Pain Medicine, Beth Israel Deaconess Medical Center, Harvard Medical School, Boston, USA; 3 Anesthesiology, Louisiana State University Health Sciences Center, Shreveport, USA; 4 Anesthesiology, Critical Care and Pain Medicine, University of Texas Health Science Center at Houston McGovern Medical School, Houston, USA

**Keywords:** low back pain (lbp), chornic pain, chronic pain management, superior cluneal nerve, peripheral nerve stimulator

## Abstract

Low back pain (LBP) is a challenging clinical condition for both patients and physicians. It requires a comprehensive initial diagnosis to avoid missing potential causes. One less common cause is superior cluneal neuralgia (SCN), which can present with limited lumbar motion, LBP, buttock pain, or an antalgic gait. While conservative therapies are often first line for LBP, neuromodulation, such as peripheral nerve stimulation (PNS), can be considered for more refractory cases. This case report is unique in that SCN was treated with a temporary PNS system, which provided sustained analgesic benefits without the need for permanent implantation.

## Introduction

Low back pain (LBP) is a challenging clinical condition for both patients and physicians. It requires a comprehensive evaluation to avoid missing potential causes. Superior cluneal neuralgia (SCN) is often an underdiagnosed contributor of chronic LBP, accounting for 1.6-14% of LBP cases [1,2]. SCN originates from the superior cluneal nerve, affecting the sensory areas of the posterior iliac crest and upper gluteus maximus [2]. Symptoms may include limited lumbar motion, LBP, buttock pain, and an antalgic gait, increasing the risk of misdiagnosis and unnecessary spinal procedures. While conservative therapies are often first line for LBP, the presence of SCN may necessitate surgical interventions [3]. More recently, neuromodulation has been considered, with permanent peripheral nerve stimulation (PNS) at the forefront [3]. This case report is unique in that SCN was treated with a temporary 60-day PNS system, which provided sustained analgesic benefits without the need for permanent implantation.

## Case presentation

A 76-year-old woman with a history of osteoarthritis and lumbar degenerative spinal stenosis presented with chronic LBP. She had undergone extensive back surgeries within the last two years, including transpedicular fixation at L2, L3, L4, and S1; sacroiliac fixation bilaterally; anterior spinal fixation and intervertebral disc cage placement at L3-L4 and L4-L5; and bilateral hip replacements. She reported non-radiating, LBP that started after her back surgeries and had been refractory to conservative and minimally invasive therapies. Physical exam findings were notable for tenderness to palpation along the superior aspect of her gluteus maximus and pain along the iliac crests. Reproduction of pain was seen with lateral bending and flexion and extension at the hip. She had tried various pharmacological treatments, including alternating acetaminophen 1000 mg every six hours with ibuprofen 800 mg every eight hours, cyclobenzaprine 5 mg three times daily, gabapentin 800 mg three times daily, and tramadol 50 mg every eight hours with minimal benefit. She had performed physical therapy, with minimal effect. Minimally invasive options, such as caudal epidural steroid injections, sacroiliac joint injections, trigger point injections with local anesthetics over the iliac crest, and lumbar medial branch blocks, offered minimal pain relief. She trialed diagnostic cluneal blocks with a mixture of 1 mL of 0.25% bupivacaine and 40 mg of triamcinolone, which provided 75% pain relief for three months.

With refractory symptoms, the patient was considering a spinal cord stimulator trial but was hesitant to pursue a permanent implantable system. Considering her options, the patient decided to pursue PNS placement at the bilateral superior cluneal nerves utilizing a temporary 60-day PNS system (SPRINT PNS System, SPR Therapeutics, Inc., Cleveland, Ohio).

Prior to the procedure, the risks and benefits were discussed, and all questions were answered. The patient was brought to the procedure room table and placed in the prone position. Routine monitors were applied, and a surgical timeout was performed. Strict sterile technique was used. The skin was prepped with a Chloraprep solution and draped in a sterile manner.

**Figure 1:**
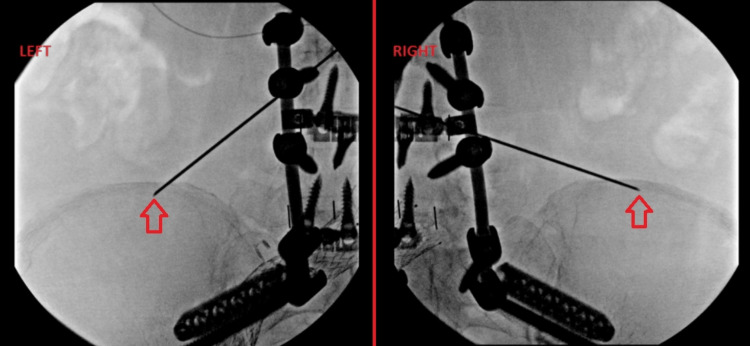
Superior cluneal nerve PNS placement. The arrows highlight the placement of the stimulating needles along the border of the iliac crest, which is the target location for the stimulation of the superior cluneal nerves.

Fluoroscopy was utilized to visualize the iliac crests. A 25-gauge, 1.5-inch needle was used to inject the skin with 2 mL of 1% lidocaine for local anesthesia a few centimeters medial and superior to the final needle placement site on each iliac crest. The stimulating needle electrodes were inserted and advanced onto the border of the iliac crest in the region of the superior cluneal nerves. Test stimulation was delivered via the stimulating test electrode to assist in identifying the optimal location for the indwelling lead. Comfortable paresthesia in the low back and buttock region confirmed appropriate placement. Two leads were then deployed to provide bilateral coverage of the superior cluneal nerves (Figure 1).

The leads were then secured with surgical glue and adhesive bandages. The patient was then brought to the recovery room and the device representative adjusted the stimulation programming to optimize therapy.

There were no complications and the patient reported progressive improvement in pain throughout her treatment. After 60 days of treatment, the leads were removed and the patient reported 80% improvement in her back pain complaints and endorsed that she was more functional and had reduced her medication utilization. She had discontinued her acetaminophen, ibuprofen, and cyclobenzaprine usage. She reduced gabapentin to 300 mg three times a day and reduced tramadol use by 75% to once a day as needed. She was seen two months after PNS lead removal and endorsed a sustained 60% pain relief with continued functional improvement and has not increased her medication usage.

## Discussion

This case demonstrates the utility of a 60-day PNS therapy for the treatment of SCN. Iatrogenic factors, such as previous iliac crest, spinal, and debridement surgeries, are common causes of SCN [1,2]. Furthermore, conditions leading to abnormal muscle tone, such as lumbar spinal stenosis, disc herniations, scoliosis, fractures, and osteoarthritis, can contribute to SCN [1,2]. Signs, such as increased tenderness, paresthesia, or allodynia along the upper part of the gluteus maximus and iliac crests, may suggest potential involvement of the superior cluneal nerve. The reproduction of pain through specific maneuvers that elongate muscles along the superior cluneal nerve pathway, such as lateral bending, stretching of the corresponding quadratus lumborum, or full flexion of the hip and knee on the same side, provides additional support for the likelihood of SCN pathology [2,4].

Conservative management with pharmacological and physical modalities is a first-line treatment option for SCN [3]. In cases resistant to these measures, local nerve blocks and ablative procedures, such as phenol neurolysis or radiofrequency ablation, may be trialed [3]. Historically, surgical decompression may be considered, targeting either the distal superior cluneal nerve branches or the involved lumbar radicular segments [3,4]. To reduce invasive therapies, previous case reports have shown that permanent PNS can provide relief for refractory SCN-induced LBP [5-7]. This case expands the option for SCN-induced LBP via a 60-day temporary PNS system, broadening the repertoire for patient care for sustained analgesic benefits compared to a permanent PNS system.

Compared to permanent implantation, the reversibility of a 60-day temporary PNS system provides the unique ability for patients to assess the effectiveness of PNS treatment before permanent implantation. If the therapy proves ineffective or if there are adverse effects, the system can be easily removed [7-9]. This patient achieved sustained relief, which could provide other patients with SCN the option to pursue temporary PNS before deciding to permanently implant.

## Conclusions

Consideration of SCN is crucial in the diagnostic evaluation of individuals presenting with LBP. Risk factors for this condition involve procedures that contribute to increased myofascial compression, such as fibrosis following spinal or gluteal interventions, along with maladaptive posturing due to pain or other medical conditions. Temporary PNS provides another viable and efficacious therapeutic strategy for SCN management that could potentially be sustained even after the removal of the device.

## References

[1] Karri J, Singh M, Orhurhu V, Joshi M, Abd-Elsayed A (2020). Pain syndromes secondary to cluneal nerve entrapment. Curr Pain Headache Rep.

[2] Karl HW, Helm S, Trescot AM (2022). Superior and middle cluneal nerve entrapment: a cause of low back and radicular pain. Pain Physician.

[3] Gill B, Cheng DS, Buchanan P, Lee D (2022). Review of interventional treatments for cluneal neuropathy. Pain Physician.

[4] Anderson D, Szarvas D, Koontz C (2022). A comprehensive review of cluneal neuralgia as a cause of lower back pain. Orthop Rev (Pavia).

[5] Chauhan G, Levy I, DeChellis D (2022). Superior cluneal neuralgia treated with wireless peripheral nerve stimulation. Cureus.

[6] Soteropoulos C, Pergolizzi J, Nagarakanti S, Gharibo C (2022). Peripheral nerve stimulation for treatment of cluneal neuropathy case study. Cureus.

[7] Abd-Elsayed A (2020). Wireless peripheral nerve stimulation for treatment of peripheral neuralgias. Neuromodulation.

[8] Hasoon J, Chitneni A, Urits I, Viswanath O, Kaye AD (2021). Peripheral stimulation of the saphenous and superior lateral genicular nerves for chronic knee pain. Cureus.

[9] Pingree MJ, Hurdle MF, Spinner DA, Valimahomed A, Crosby ND, Boggs JW (2022). Real-world evidence of sustained improvement following 60-day peripheral nerve stimulation treatment for pain: a cross-sectional follow-up survey. Pain Manag.

